# Fertility preservation in 2026: current landscape in Europe and the United States for children and adolescents undergoing hematopoietic stem cell transplantation—recommendations on behalf of the Westhafen Intercontinental Group

**DOI:** 10.1038/s41409-026-02861-w

**Published:** 2026-05-12

**Authors:** Andrea Jarisch, Seth J. Rotz, Alessandro Cattoni, Blandine Courbiere, Scott D. Lundy, Kyle E. Orwig, Catherine Poirot, Jean-Hugues Dalle, Rachel Phelan, Tamara Diesch-Furlanetto

**Affiliations:** 1 Division of Pediatric Stem Cell Transplantation and Immunology, Department of Children and Adolescents, Frankfurt University Hospital, Goethe University Frankfurt, Frankfurt am Main, Germany; 2 EBMT Pediatric Diseases Working Party, Paris, France; 3 Pediatric Hematology Oncology and Blood and Marrow Transplantation, Cleveland Clinic, Cleveland, OH USA; 4 Pediatric Department, Fondazione IRCCS San Gerardo Dei Tintori, Monza, Italy; 5 Department of Gynecology-Obstetric and Reproductive Medicine, AP-HM, Hôpital La Conception, Marseille, France; 6 IMBE, Aix-Marseille Univ, Avignon Univ, CNRS, IRD, Marseille, France; 7 Glickman Urological Institute, Cleveland Clinic, Cleveland, OH USA; 8 Department of Obstetrics, Gynecology and Reproductive Sciences, Magee-Womens Research Institute, University of Pittsburgh School of Medicine, Pittsburgh, PA USA; 9 Hematology for Adolescents and Young Adults, Hôpital Saint Louis, GHU AP-HP Nord Université Paris Citè, Paris, France; 10 GRECOT (Groupe de Recherche et d’Ètude sur la Cryoconservation de l’Ovaire et du Testicule), Paris, France; 11 Robert-Debré Academic Hospital, GHU AP-HP Nord Université Paris Citè, Paris, France; 12 CIBMTR ® (Center for International Blood and Marrow Transplant Research), Medical College of Wisconsin, Milwaukee, WI USA; 13 Division of Pediatric Hematology/Oncology/Blood and Marrow Transplant, Department of Pediatrics, Medical College of Wisconsin, Milwaukee, WI USA; 14 Department of Pediatric Hematology/Oncology, University Children’s Hospital of Basel, Basel, Switzerland

**Keywords:** Paediatrics, Risk factors

## Abstract

Advances in hematopoietic stem cell transplantation (HSCT) and other cellular therapies have continued to improve the long-term outcomes for pediatric patients undergoing these treatments. However, treatment impacts on future fertility can have a substantial impact on the patient's quality of life. Concurrent with improvements in transplantation, fertility preservation (FP) options and outcomes have also evolved. Options such as ovarian tissue cryopreservation and testicular tissue cryopreservation are now more widely available and can be considered for very young patients. The timing and choice of FP to be offered can be complex, and many considerations must be made when counseling patients and families. Cost and access are also variable depending on the country and region in which a patient resides. The aim of this paper is to provide an updated overview of the current strategies and recommendations for FP that should be offered to all children and adolescents undergoing an allo-HSCT. By summarizing contemporary evidence and practices, this paper may provide support to clinicians in delivering equitable, informed, and accessible FP counseling and care for all eligible patients.

## Introduction

Allogeneic hematopoietic stem cell transplantation (HSCT) is a highly effective and potentially curative therapy in pediatric patients, applicable to high-risk malignant hematological disorders as well as severe non-malignant conditions, including hemoglobinopathies, inborn errors of immunity, aplastic anemia, and specific metabolic diseases [1, 2]. Autologous HSCT serves as a consolidation therapy for several poor-prognosis solid tumors, such as N-myc–amplified and/or stage IV neuroblastoma and certain brain tumors [1, 3]. Moreover, hematopoietic stem cell–based gene therapy has recently expanded therapeutic options for inherited diseases, such as leukodystrophies and hemoglobinopathies [4].

Approximately 75–80% of all pediatric allogeneic HSCTs, as well as nearly all autologous HSCTs and gene therapy procedures, employ myeloablative conditioning regimens that combine either total body irradiation (TBI) or alkylating agents [5]. These regimens are associated with a high risk of gonadal damage in both male and female patients, posing a serious threat to future fertility [6]. The individual risk must be assessed comprehensively, considering prior treatments, the specific composition and intensity of the conditioning regimen, the underlying disease, as well as the patient’s sex, age, and pubertal stage.

Fertility impairment remains one of the principal long-term adverse consequences impacting quality of life in HSCT survivors. Therefore, comprehensive and transparent counseling regarding fertility risks and available preservation options is essential and, in several countries, legally required.

This paper aims to provide an updated overview of current strategies for FP in the pediatric HSCT setting and includes recommendations from a subgroup of the Westhafen Intercontinental Group, which is comprised of leaders from the Center for International Blood and Marrow Transplant Research (CIBMTR) Morbidity, Recovery and Survivorship Working Committee and the Pediatric Diseases Working Party of the European Society for Bone and Marrow Transplantation (EBMT). Consensus was achieved through representatives from each of the groups that were tasked with paper planning, writing, and editing. By summarizing contemporary evidence and practices and explicitly recommending the implementation of FP measures, we seek to support clinicians in delivering equitable, informed, and accessible FP counseling and care for all eligible patients.

## Counseling and decision making

Providing fertility counseling during the pre-HSCT phase is challenging for transplant physicians. While having to deliver concrete facts and figures about HSCT-related risks, they must also honor the patient’s autonomous wishes, ideas and individual judgments to guarantee certified informed consent, in the specific triangular pediatric setting. Patients and their parents must be informed about the indication for transplantation, the procedures involved, the acute side effects, the expected long-term outcomes, and the risk of death. Mentioning possible long-term side effects, including fertility issues, often increases concerns about the procedure, even though it suggests long-term survival. Among the information received pre-transplant, the risk of infertility can be amongst the most devastating [7].

Various FP options can be tailored to patients and available resources [8]. It is crucial to provide clear information about standard and experimental options for preservation. Obtaining informed consent is mandatory for all FP procedures, with special attention for minors. This requires parental consent and, whenever possible, the child’s assent. When experimental FP techniques are considered, their investigational nature, potential risks, and uncertainties must be clearly explained, and such interventions should only be offered under approved clinical research protocols. Several conditions for which transplant is offered may have specific procedural concerns that patients and clinicians should consider (Table 1). The concepts of fertility, childbearing and parenthood are strongly influenced by culture [9]. Therefore, an individual’s opinions on semen preservation, masturbation, electroejaculation and ovarian or testicular biopsies may differ greatly depending on ethnic and cultural background.

**Table 1:** Restrictions on fertility preservation measures in special situations.

Ref	Disease	Special Considerations
[104, 121–123]	Red cell disorders TDT, SCD, BMFS	—Hypogonadism due to transfusion-related iron overload
[104, 121, 123–128]	SCD	—Gonadotoxic influence of HU: general recommendation of >2 months off-HU, prior preservation measures —Ovarian Stimulation: Possible hormone-related complications (severe pain crisis, occurrence of thromboses, especially acute thoracic syndrome or central nervous system infarctions); OTC may be preferred —Anesthetic and disease-specific precautions (e.g., fluids, transfusion prior to anesthesia)
[123]	BMFS, MDS-RCC, SAA	—Thrombocytopenia/Neutropenia: increased risk of bleeding or infection
[123]	Genetic Disorders	—Transmission of genetic disorder should be accounted for/mentioned in counseling; consider involvement of a licensed genetic counselor —Disease-related fertility limitations

*BMFS* Bone Marrow Failure Syndrome, *FP* Fertility Preservation, *HU* Hydroxyurea, *MDS-RCC* Myelodysplastic Syndrome-Refractory Cytopenia of Childhood, *SAA* Severe Aplastic Anemia, *SCD* Sickle cell disease, *TDT* Transfusion dependent thalassemia.

In children, HSCT is often used as treatment for genetic diseases. When discussing fertility-preserving measures, the possible transmission of underlying diseases, especially X-linked diseases, should be considered and mentioned in the counseling interview. Genetic counselling should be proposed in parallel.

Counselling should include the following [1]. An infertility risk assessment, considering the patient’s underlying disease, age, pre-SCT treatment and planned conditioning, as well as any known co-morbidities that may impact fertility regardless of HSCT [2]. An overview of established and experimental FP techniques, tailored to the patient’s sex, age, and underlying disease [3]. An in-depth discussion with the patient and their family about the most suitable preservation strategies, as well as a discussion of the risks and side effects associated with the procedure and the prospects and costs for fertility restoration.

It is strongly recommended to offer counselling related to FP opportunities to every patient receiving HSCT as part of the pre-SCT workup. Psychological support should accompany counseling, as FP decisions can be emotionally complex. Comprehensive care is best delivered through a multidisciplinary team including pediatric oncologists/hematologists, nurse navigators, reproductive specialists, endocrinologists, and psychologists.

## Fertility preservation techniques: biological and medical considerations

### Pre-Pubertal FP

#### Females

**Fig. 1:**
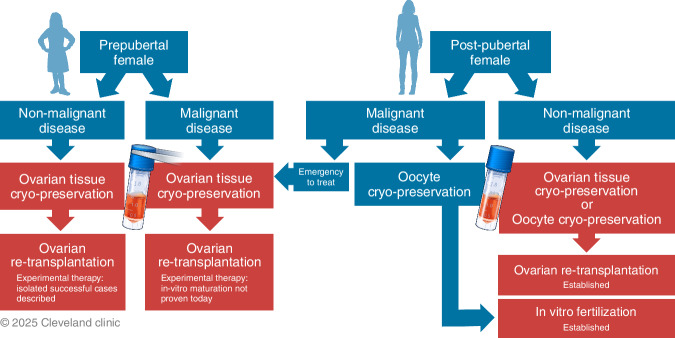
**Various FP options exist for females depending on menarchal status, HSCT indication (i.e., malignant vs. non-malignant), available time for FP prior to starting gonadotoxic therapies, and social circumstances (i.e., does the patient have a partner they wish to cryopreserve embryos with rather than oocytes alone).** In pre-menarchal females, gonadal tissue cryo-preservation is the only available option as the patient is not yet able to ovulate. In both pre- and post-menarchal females with malignancies who have undergone OTC, the decision to re-implant tissue is based on the reported risk of occult malignant cell contamination of the ovary (although very rarely reported to result in relapsed disease) as well as the potential for future ex vivo oocyte maturation (currently considered experimental). Ovarian tissue reimplantation also has the potential to temporarily restore hormone function.

For prepubertal girls, ovarian tissue cryopreservation (OTC) is currently the only available FP option, as they do not yet produce mature oocytes (Fig. 1). The procedure involves laparoscopic removal of ovarian tissue, which is subsequently dissected and cryopreserved. Depending on the underlying disease, this tissue can be transplanted back either orthotopically or heterotopically to restore both endocrine and exocrine ovarian function and fertility, though the half-life of the reimplanted ovarian tissue is limited to a few years. OTC has resulted in live births from cryopreserved tissue from adults and adolescents [10–14]. However, outcome data from tissues stored for prepubertal patients remain limited due to the time between tissue harvesting and potential use [15]. Experimental options, such as in vitro folliculogenesis to obtain mature oocytes from prepubertal ovarian tissue, are under active investigation but are not yet clinically established [16–18].

#### Males

**Fig. 2:**
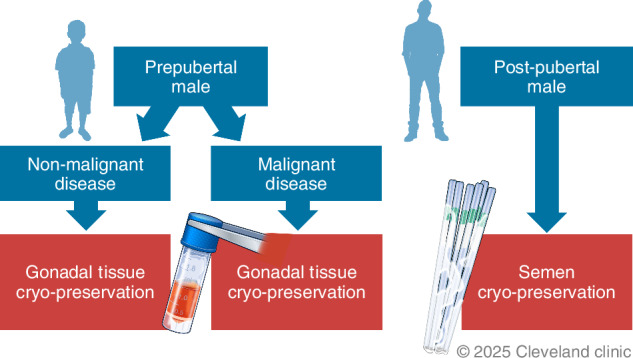
**For prepubertal males, TTC is presently offered at some institutions on a research protocol basis, while a few centers may offer it routinely.** Semen cryopreservation can be quickly completed for most patients and is the standard of care for post-pubertal males. In rare circumstances, TTC in post-pubertal males may be considered.

For prepubertal males, testicular tissue cryopreservation (TTC) represents the only FP strategy, as spermatogenesis has not commenced (Fig. 2) [19]. The procedure entails surgical biopsy of testicular tissue, which is then cryopreserved. Potential future applications include auto-transplantation of spermatogonial stem cells/tissue or in vitro maturation of spermatogonia (cells); however, all of these approaches remain experimental in humans [20–24]. To date, no pregnancies have been reported in humans using TTC, although restoration of spermatogenesis and live births have been achieved in animal models [23–26].

### Post-pubertal fertility preservation

#### Females

##### Oocyte Cryopreservation

Post-pubertal females may undergo oocyte cryopreservation, which involves hormonal stimulation to induce the growth of mature follicles. Oocyte retrieval is performed transvaginally under ultrasound guidance, followed by vitrification for storage. When a future pregnancy is desired, thawed oocytes are fertilized with sperm via in vitro fertilization (IVF) or intracytoplasmic sperm injection (ICSI). Reported live birth rates per thawed oocyte range from 4% to 25% [27, 28], thus clinicians aim for collecting as many oocytes as feasible. Time is an important consideration. Some patients may need to proceed immediately to treatment and do not have the 2–3 weeks that are required for hormonal stimulation and collection.

#### Embryo cryopreservation

Embryo cryopreservation involves ovarian stimulation and oocyte retrieval as described above, followed by fertilization using partner or donor sperm. The resulting embryos are then cryopreserved. In future pregnancy attempts, thawed embryos are transferred into the uterus. Live birth rates per thawed embryo are reported between 30% and 50% [28, 29]. This option is typically not feasible for pediatric/adolescent patients due to their age and relationship status. Similar to oocyte cryopreservation, time is a consideration. For patients who do not have time for oocyte or embryo cryopreservation, ovarian tissue cryopreservation can sometimes be quicker achieved.

#### Ovarian tissue cryopreservation

In post-pubertal females, OTC follows the same procedure as in prepubertal girls, and can be undertaken rapidly if the patient is safe for anesthesia and surgery. OTC is indicated for patients who are unable to undergo ovarian stimulation or require urgent initiation of gonadotoxic therapy and before highly gonadotoxic treatments. Several hundred live births have been reported (and likely many more not published), and ~30% who have ovarian tissue reimplanted give birth [30–34].

#### Gonadotropin-releasing hormone agonists (GnRHa)

Gonadotropin-releasing hormone agonists are administered before and during chemotherapy with the aim of suppressing ovarian function and thereby reducing chemotherapy-induced damage [35].

However, evidence regarding their efficacy for FP is debated [36]. The last guidelines of ASCO underlined that GnRHa should not be used in place of established FP methods [37].

#### Males

##### Semen Cryopreservation

In post-pubertal males, semen cryopreservation should be done before treatment, even when needing to start therapy emergently. Semen parameters of patients undergoing treatment may be inferior to those of healthy controls due to their underlying condition [38]. At times, cryopreserving multiple samples may be beneficial, if feasible. If there are no sperm in the ejaculate, it may still be possible to retrieve sperm directly from the testes by testicular sperm extraction (TESE) [39, 40].

### Treatment-related toxicity and fertility impact

Conditioning regimens administered before HSCT are intrinsically gonadotoxic, leading to profound long-term reproductive endocrine sequelae [41, 42]. These effects are highly dependent on the type and dose of conditioning, and the patient’s age and sex [6, 43]. Understanding the differential impact on gonadal function in males and females is critical for developing FP strategies and optimizing survivorship care; inherent challenges in understanding fertility risk make this task [37].

#### Females

In females, gonadal impairment manifests along a spectrum ranging from the subclinical “diminished ovarian reserve” (DOR) to overt premature ovarian insufficiency (POI). DOR, characterized biochemically by low anti-Müllerian hormone (AMH) levels and reduced antral follicle count, retains menstrual cyclicity but indicates a diminished follicular pool with compromised fertility potential. In the setting of POI, the combination of amenorrhea, hypoestrogenism and elevated gonadotropins implies extensive follicular depletion, leading to severe endocrine dysfunction along with profound fertility impairment [44, 45].

Overall, the reported incidence of POI following HSCT in childhood ranges from 44 to 100% [43, 46–49]. This variability reflects differences in the gonadotoxicity of conditioning regimens and demographic diversity. Research into fertility potential is complicated by the long follow-up periods required, as well as a lack of data on patients’ desires for biological parenthood (Table 2). Most studies report pregnancy rates without distinguishing between those who do and do not wish to conceive [50, 51]. While AMH is a useful biomarker in female survivors, it has important limitations [52, 53].

**Table 2:** Studies of Fertility Potential Among HCT Survivors.

Ref	Sex	Disease/ Population	Outcome Measure	Findings	Limitations
[64]	Female	Age <40 at time of HCT, all diseases	AMH 1-2 years post-HCT	—1/ 45 (2.2%) MAC v. 4/26 (15.4%) RIC AMH ≥ .5 ng/m (*P* = 0.06) —8/45 (17.8%) MAC, 12/26 RIC (46.2%) detectable AMH (*P* = 0.015) —TBI and CED not associated with detectable AMH —AMH not detectable for CED between 0 and 26.600 mg/m ^2^; AMH detectable for CED between 0 and 13580 mg/m^2^	—Small sample size
[50]	Both	Age <18 at time of HCT, all diseases	Pregnancy, pregnancy outcome	—7/7 males conceiving, who received TBI used cryopreserved semen or required assisted fertilization techniques —13/25 (52%) females conditioned with TBI, 50/52 (96%) conditioned w/out TBI conceived naturally —63/7- (90%) female conceptions resulted in live birth	—limited follow-up length for a pediatric cohort, relied upon surveys being returned (response bias)
[129]	Both	Female HCT survivors who became pregnant and males who sired a pregnancy, malignant +non-malignant, auto+ allo	Pregnancy	—live birth in 85% of pregnancies —9/82 pregnant women received TBI ≥ 400 cGy —18/95 men received TBI ≥ 400 cGy	—use of cryopreserved embryos, semen, and donor oocytes, not collected
[108]	Both	All patients undergoing HCT, all ages, auto+ allo	Pregnancy, live birth	—0.6% conceived after HCT —higher rates of C-section and preterm delivery compared to the general population	—presented as % of all transplanted patients (regardless of age, survival), not those desiring pregnancy —included patients with cryopreserved sperm, embryos
[130]	Female	—remission post-HCT and were alive for at ≥1 yr	AMH	—detectable AMH levels 18/80 (23%), of whom 12/80 (15%) AMH ≥ 0.5 ng/m —of 18 detectible patients, 16 received RIC, 12 pre-menarchal at HCT	—various timepoints, small sample size
[131]	Female	—age 18-40, allo	Pregnancy, live birth	—50/2654 became pregnant (74 pregnancies overall) —57/74 pregnancies resulted in live birth —younger age at HCT, nonmalignant disease indications, no TBI or a cumulative dose <8 Gy, and nonmyeloablative/ RIC	—Several pregnancies occurred among women using cryopreserved or donor material; information on cryopreserved/donor material use is not available for all —unknown pregnancy desire
[132]	Both	Underwent alloHCT with Treosulfan/ Fludarabine regimen +/-200cGy TBI	Pregnancy	—18/345 patients or partners achieved pregnancy without assisted methods	—no comparator group, unknown pregnancy desire
[74]	Both	Children undergoing single alloHCT, surviving >1 year	AMH (F), Inhibin B (M)	—higher AMH levels in treo regimens vs. Flu/Mel or Bu/Cy —higher Inhibin B levels in treo regimens vs. Flu/Mel or Bu/Cy	—treo group youngest at HCT —mostly non-malignant patients
[65]	Female	Underwent allo-HCT at <21 years old; Sickel Cell Disease	AMH 2 years post-HCT	—15/17 had undetectable AMH post-HCT —1/17 with low, detectible AMH (Alemtuzumab/Flu/Mel (CED = 5,600) —1/17 with low, detectible AMH (Flu/ Cy/ TT/ ATG/ 200 cGy TBI (CED = 18,750) —14/18 received <8,000 CED. The 3 remaining patients received CED included between 17.600 and 18.700 mg/m^2^	—small sample size —single HCT indication
[66]	Both	Aplastic anemia, allo-HCT, in a relationship desiring pregnancy, survived ≥2 years post-HCT; Flu/Cy or Cy/ATG conditioning	Pregnancy	—82% became pregnant or sired pregnancy —no difference in Cy/ATG vs. Flu/Cy —older age, cGVHD associated with a lack of fertility	—no data on assisted techniques or cryopreservation

*allo* allogeneic, *AMH* Anti-Mullerian Hormone, *ATG* Anti-Thymocyte Globulin, *Bu* Busulfan, *cGVHD* chronic Graft-versus-Host Disease, *CED* Cyclophosphamide Equivalent Dose, *Cy* Cyclophosphamide, *Flu* Fludarabine, *MAC* Myeloblastic Conditioning, *Mel* Melphalan, *RIC* Reduced Intensity Conditioning, *TBI* Total Body Irradiation, *treo* treosulfan, *TT* Thiotepa.

Both alkylating agents and TBI involve depletion of ovarian reserve in a dose-dependent manner [54]. The gonadotoxicity of conditioning regimens correlates directly with cumulative doses. Biologically, TBI-related gonadotoxicity results from radiation-induced DNA strand breaks, leading to massive follicular apoptosis. Radiation exposure exceeding 10 Gy has been demonstrated to result in POI with occurrence as high as 95–100% among post-pubertal acute myeloid leukemia transplanted survivors [54, 55]. Younger age upon radiation exposure is regarded as protective. Although 40–60% of prepubertal girls <10 years at the time of transplantation experience a progression of puberty and spontaneous menarche, the risk of later-onset POI remains remarkable following TBI. As TBI is never administered as the only treatment, drawing a sterilizing radiant threshold can be cumbersome. Mathematic modeling based on data from rates of oocyte decline has set the following age-dependent thresholds for the risk of developing POI: 20.3 Gy in infants, 18.4 Gy at the age of 10 years, and 16.5 Gy at the age of 20 [56]. However, gonadal doses much lower are associated with a substantial reduction in fertility potential [57, 58].

Among alkylating agents, busulfan is universally acknowledged as the most gonadotoxic, with POI occurrence rates exceeding 90% following standard-dose administration [59, 60]. On the other hand, a greater percentage of retained ovarian function has been reported following cyclophosphamide- and melphalan-based conditioning regimens [55, 61].

In order to compare the cumulative burden of gonadal toxicity following polychemotherapy protocols and to compare regimens, the cyclophosphamide equivalent dose (CED) method has been developed [62]. By applying dedicated coefficients, CED allows clinicians to estimate the overall alkylating dose administered, expressed as an equivalent dose of cyclophosphamide. The CED was derived from comparisons of hematological toxicity across various alkylating agents, so its application to ovarian function should be interpreted with caution” [63]. However, recent studies have shown even among patients with low-moderate risk of infertility based on cumulative CED alone [57], the vast majority of girls undergoing HSCT had very low or undetectable levels of AMH [64, 65]. Alternatively, in a single-center study of patients with aplastic anemia receiving cyclophosphomide (at lower cumulative doses) as the only alkylating agent, the majority were able to achieve pregnancy [66]. Recently, a growing body of literature has highlighted the gonadal-sparing profile of treosulfan, with lower occurrence of POI and need for pubertal induction among patients conditioned at different ages and baseline pubertal status [67]. A conversion factor for Treosulfan CED has yet to be established.

### Males

In males, germ cells are more radio- and chemo-sensitive than Leydig cells, and the prevalence of azoospermia following HSCT exceeds 85 to 90% in cohorts of patients transplanted for hematological malignancies or bone marrow failure utilizing myeloablative conditioning regimens [68]. Conversely, testosterone secretion is generally retained following chemo-only regimens [69] while up to half of cancer survivors experience hypogonadism after TBI [70, 71].

The probability and severity of testicular damage are dose-dependent. Based on mathematical models, the radiant sterilizing dose is generally set between 2 (adults) and 6 Gy (pre-pubertal patients). In terms of the detrimental effect of alkylating agents, spermatogonial cell depletion and subsequent oligo-azoospermia frequently occur for busulfan and for cyclophosphamide doses >7.5–9 g/m^2^ [72, 73]. More recently, a testicular-sparing profile has been hypothesized among patients conditioned with treosulfan [69, 74].

In male survivors, biomarkers beyond formal semen analysis are of limited reliability [75].

### Access and cost coverage

FP is now recognized as an essential part of care for pediatric patients undergoing HSCT or other cellular therapies. Despite increasing awareness, access, and implementation remain highly variable across centers.

**Fig. 3:**
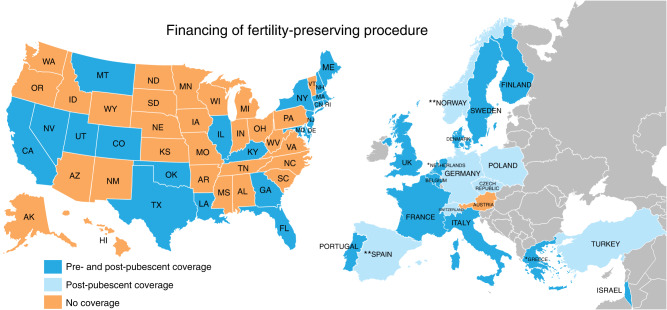
**Insurance coverage for various FP procedures varies by state in the United States and by country in Europe.** In the United States, individual state laws mandate different degrees of insurance coverage for FP procedures, and significant variability exists between what is and is not covered. Additionally, most states do not mandate individual insurance coverage, and therefore some patients will not have any insurance coverage regardless of state insurance laws. *Coverage for FP procedures but not storage. **Coverage for pre-pubertal ovarian tissue cryopreservation, but not for testicular tissue cryopreservation.

Differences stem from national legislation, insurance coverage, institutional resources, technical capacity, and awareness among providers and families. Some countries, such as France and Israel, have legally mandated access to FP, whereas others rely on institutional or regional guidelines, leading to significant disparities (Fig. 3).

Networks like FertiPROTEKT, the Danish Network, the Oncofertility Consortium (www.oncofertility.northwestern.edu), Alliance for Fertility Preservation (www.allianceforfertilitypreservation.org), Orchid-Net, and the French National Institute (www.cancer.fr) promote standardization and knowledge sharing, supported by organizations such as ESHRE and PanCareLIFE [19, 25, 76–78]. In the U.S., the Children’s Oncology Group and the American Society for Reproductive Medicine (ASRM) have issued similar guidelines to expand equitable FP access [79–81].

Although financial support has improved, major gaps persist—particularly for prepubertal patients and in regions without national coverage. The authors herein advocate for universal access to FP counseling and funding for all pediatric HSCT patients to ensure equity in fertility care.

### Implementation and timing of fertility preservation

The timing of FP in children and adolescents referred for HSCT depends on balancing several key factors. Early counseling and referral to reproductive specialists are essential, ideally at diagnosis, when HSCT is a likely therapeutic path. A disease-specific, individualized approach should identify possible “windows of opportunity” at diagnosis, between treatment cycles, in remission, or just before conditioning (Table 3).

**Table 3:** Considerations on Timing and Feasibility of Fertility Preservation.

Question	Consideration
**Pre-treatment**	
Can FP be completed at diagnosis?	—Ideal, if time allows —urgent need for disease control may preclude —anesthesia may not be feasible due to acute concerns (e.g., large mediastinal mass) —Although technically feasible, acute illness may decrease the quality of collection [133].
Is there a risk of malignant contamination of cryopreserved tissue?	—largest concern in leukemia, other diseases also have a theoretical risk —future ability to perform ex vivo maturation of tissue
**During treatment**	
Has the patient recently received gonadotoxic/genotoxic chemotherapy?	—Variable data on genotoxicity and risk to potential offspring —Most centers recommend >3 months since last chemotherapy before collection of mature oocytes or sperm –If there is no other time-window or option, what risk is the patient willing to accept –Prior treatment drug and dose should be considered –Ovarian or testicular cryopreservation can be performed after the start of chemotherapy.
**Post-treatment**	
Should a survivor who did not initially undergo FP be offered FP?	–Consider if residual gonadal function is present –Should subsequent collections be completed (i.e., additional sperm, oocyte)? What is the realistic chance of successful fecundity with the present material? —Should a potentially more successful technique be used at a later point (i.e., if a prepubescent male had TTC, and is now able to produce sperm but is at high risk of relapse)? —Refer to a fertility specialist to help determine fertility potential; it may take months to years to recover post-chemotherapy.

At various time-points in a patient’s treatment, FP should be re-examined and potentially rediscussed. Some patients have an existing, but often shortened, reproductive window after HSCT [82]. If primary FP was not performed, was ineffective, or done in prepubertal patients before gonadotoxic treatment, secondary FP should be considered approximately 1 year after treatment or after entering puberty [83].

### Risk of malformation following chemotherapy exposure

Children born via spontaneous conception to fathers who underwent chemotherapy or radiotherapy years prior do not demonstrate a significant increase in the risk of congenital malformations compared to the general population [84, 85]. Patients should be advised of potentially higher genetic damage risks in sperm collected immediately post cancer-directed therapy [37].

Similarly, children of mothers who are cancer survivors and received chemotherapy or radiotherapy prior to conception do not appear to have an increased malformation risk compared to the general population [86].

### Risk of malformation after fertility preservation

Data concerning the risk of congenital malformations following FP are scarce, and long-term cohorts of women who conceive after returning vitrified oocytes or frozen-thawed ovarian tissue for cancer are needed. In a large registry cohort of singleton livebirths, children conceived without medical assistance had a 4.2% incidence of major malformation. In the context of assisted reproductive technologies, this risk is 6% after fresh intracytoplasmic sperm injection(ICSI), 5.3% conceived using fresh in vitro fertilization(IVF), and 4.9% of children conceived using cryo-ICSI [87].

Pregnancies from vitrified oocytes demonstrate no significant rise in anomaly rates compared with fresh oocytes, with reported incidences around 1–2% [88]. Although data on offspring from ovarian tissue reimplantation (OTT) after OTC remain limited, especially in those who underwent OTC during childhood, no clear evidence suggests an excess risk compared to spontaneous or ART conceptions [89]. Similarly, sperm cryopreservation, while potentially affecting sperm function or structure, has not been associated with higher congenital anomaly rates [90].

### Risk of malignant cell reintroduction

Reimplantation of cryopreserved gonadal tissue carries a potential risk of malignant cell reintroduction, particularly in diseases with bone marrow involvement. Collecting tissue during complete remission reduces this risk, though the optimal threshold—based on bone marrow morphology, flow cytometry, or next-generation sequencing—remains undefined [91–93]. Minimal residual disease has been detected in both ovarian and testicular tissue from leukemia patients [92–94].

Despite these concerns, several successful OTT after leukemia have been reported, with only one relapse reported [95–100]. Harvesting post-chemotherapy tissue may further lower contamination risk [101]. Laboratory decontamination protocols and in vitro procedures from cryopreserved tissue are emerging research strategies to restore fertility while avoiding reimplantation of malignant cells [102, 103].

### Special considerations

#### Gene therapy

Emerging treatments using genetically modified hematopoietic stem cells show encouraging outcomes for non-malignant disorders such as leukodystrophies, immunodeficiencies, and hemoglobinopathies. Current protocols typically include myeloablative conditioning with alkylating agents. Fertility counseling before therapy should include discussion of the risk of inherited transmission of the underlying genetic disease, discuss available preservation options and timing, and consider specific factors such as anesthesia safety, iron overload, and discontinuation of hydroxyurea [104].

#### Chimeric antigen receptor T cell therapy

Assessing fertility risk from CAR T-cell therapy alone is difficult, as most patients also receive gonadotoxic agents like cyclophosphamide for lymphodepletion. Despite this, live births have occurred following CAR T-cell therapy [105]. Potential fertility effects may arise from inflammatory or immune-mediated mechanisms [106]. Theoretical risks include transplacental passage of CAR T-cells, possibly affecting fetal B-cell function [105, 107].

#### Uterine dysfunction following TBI or busulfan exposure

Along with gonadal insufficiency, uterine function can be affected following HSCT. Irradiation may damage the uterus and compromise fertility irrespective of ovarian insufficiency. In addition, patients who conceive following TBI show remarkably greater rates of pregnancy and birth complications, including abortion, preterm delivery and low-birth weight [108, 109]. Abnormal uterine function following TBI is an integrated effect of prolonged hypoestrogenism *plus* direct radiation-induced changes [110]. The latter includes impaired uterine vascularization, iatrogenic loss of elasticity and negative effect on endometrial health and embryo receptivity [111, 112]. Younger age upon exposure results in smaller uterine volumes in adulthood, supporting the hypothesis of the detrimental role of TBI on early organ development [113].

Although TBI has been identified as most damaging, uterine volume may also be negatively affected by alkylating agents. A case-control study involving 88 transplanted patients from 13 French Centers showed alkylating agents- and TBI-based conditioning regimens were associated with a 43% and 75% reduction of uterine volume compared to healthy women, respectively, despite adequate hormonal replacement therapy [114].

Accordingly, pregnant women who have undergone HSCT deserve dedicated pre-conceptional counselling and close gynecological follow-up, in case of complications arising over gestation. A study recently compared the pregnancy outcomes of 86 women who conceived following HSCT for pediatric ALL *versus* 180 patients exposed to conventional chemotherapy [115]. Despite superimposable outcomes between alkylating-based conditioning regimens and conventional chemotherapy, TBI was associated with higher occurrence rates of miscarriage (24% *versus* 3%), preterm delivery (50% *verses* 5%), intrauterine growth restriction (21% *versus* 5%), and postpartum hemorrhage (31% *versus* 10%).

#### Gonadal insufficiency risks post-fertility preservation

Results are conflicting, and long-term data about ovarian reserve following OTC are lacking. In a study published by the French L.E.A network on ovarian function outcomes among patients treated with HSCT before puberty, unilateral oophorectomy for OTC was associated with an increased risk of POI [116]. However, these conclusions are drawn from a limited sub-cohort of only 13 patients. In addition, there was a statistically significant association between exposure to OTC and older age at HSCT, a universally acknowledged independent risk factor for ovarian damage. Khan et al. suggested a compensatory mechanism involving the contralateral ovary, as women with a single ovary who underwent IVF show a higher number of oocytes retrieved as compared to the ipsilateral ovary in women with bilateral ovaries [117]. Assessing the burden of unilateral oophorectomy on the timing of menopause onset is hampered by conflicting published outcomes. While epidemiological models estimate that unilateral oophorectomy anticipates the onset of menopause only by one year [118, 119], Teinturier and colleagues demonstrated a 7-year anticipation among cancer survivors [120].

In contrast, hemi-oophorectomy or cortical biopsies preserve more ovarian tissue in situ, but the remaining cortex could be exposed to the surgery-related damage and to gonadotoxic treatments. Plus, the overall amount of cortical tissue available for future reimplantation is reduced compared to whole-ovary withdrawal.

### Fertility preservation in countries with growing transplant activity but limited prior FP experience

Hematopoietic cell transplantation, particularly with PTCy-based approaches, is expanding in regions such as the Middle East, Africa, and Southeast Asia. Despite this growth, access to fertility preservation (FP) services in many of these areas remains limited. Establishing partnerships with countries that have established FP programs would help ensure that patients in these regions can benefit from appropriate FP options.

## Conclusion

FP has become an integral component of comprehensive care for children and adolescents undergoing HSCT. Advances in cryopreservation, surgical techniques, and multidisciplinary counseling have significantly improved the prospects for reproductive health among survivors. However, access, timing, and implementation remain inconsistent across institutions and countries, reflecting variations in resources, legislation, and awareness.

To achieve equitable care, standardized international guidelines, structured referral pathways, and financial coverage must be universally adopted. Continued collaboration among transplant physicians, FP specialists, and policymakers will be essential to ensure that every pediatric patient facing HSCT is offered timely counseling and the opportunity for FP, regardless of geographic or socioeconomic barriers.

## References

[1] Passweg JR, Baldomero H, Peters C, Gaspar HB, Cesaro S, Dreger P, et al. Hematopoietic SCT in Europe: data and trends in 2012 with special consideration of pediatric transplantation. Bone Marrow Transpl. 2014;49:744–50. 10.1038/bmt.2014.55. 10.1038/bmt.2014.55 PMC4051369 24637898

[2] Spellman SR, Xu K, Oloyede T, Ahn KW, Akhtar O, Bolon YT, et al. Current activity trends and outcomes in hematopoietic cell transplantation and cellular therapy - a report from the CIBMTR. Transpl Cell Ther. 2025;31:505–32. 10.1016/j.jtct.2025.05.014. 10.1016/j.jtct.2025.05.014 PMC12302970 40398621

[3] Passweg JR, Baldomero H, Ciceri F, de la Cámara R, Glass B, Greco R, et al. Hematopoietic cell transplantation and cellular therapies in Europe 2022. CAR-T activity continues to grow; transplant activity has slowed: a report from the EBMT. Bone Marrow Transpl. 2024;59:803–12. 10.1038/s41409-024-02248-9 PMC11161408 38438647

[4] Locatelli F, Cavazzana M, Frangoul H, Fuente J, Algeri M, Meisel R. Autologous gene therapy for hemoglobinopathies: from bench to patient’s bedside. Mol Ther. 2024;32:1202–18. 10.1016/j.ymthe.2024.03.005. 38454604 10.1016/j.ymthe.2024.03.005 PMC11081872

[5] Nagler A, Shimoni A. Conditioning. In: Carreras E, Dufour C, Mohty M, Kroger N (editors). The EBMT handbook: hematopoietic stem cell transplantation and cellular therapies. 7th edn. Cham: Springer; 2019. pp. 99–107. 32091673

[6] Vidal A, Bora C, Jarisch A, Pape J, Weidlinger S, Karrer T, et al. Impact of haematopoietic stem cell transplantation for benign and malignant haematologic and non-haematologic disorders on fertility: a systematic review and meta-analysis. Bone Marrow Transpl. 2025;60:645–72. 10.1038/s41409-025-02520-6. 10.1038/s41409-025-02520-6 PMC12061765 40074785

[7] Dalle JH, Lucchini G, Balduzzi A, Ifversen M, Jahnukainen K, Macklon KT, et al. State-of-the-art fertility preservation in children and adolescents undergoing haematopoietic stem cell transplantation: a report on the expert meeting of the Paediatric Diseases Working Party (PDWP) of the European Society for Blood and Marrow Transplantation (EBMT) in Baden, Austria, 29-30 September 2015. Bone Marrow Transpl. 2017;52:1029–35. 10.1038/bmt.2017.21. 10.1038/bmt.2017.21 28287638

[8] Barnbrock A, Hamannt F, Salzmann-Manrique E, Rohm T, Lange S, Bader P, et al. Look at the future-perceptions of fertility counseling and decision-making among adolescents and their parents in the context of hematopoietic stem cell transplantation—experience of one major center for pediatric stem cell transplantation. Front Pediatr. 2023;11:1249558. 38094191 10.3389/fped.2023.1249558 PMC10716475

[9] Latif S, Davies M, Vaughan E, Mavrelos D, Lavery S, Yasmin E. Clinical and ethical perspectives of ovarian stimulation and oocyte cryopreservation in adolescents: 6 years experience from a tertiary centre. Hum Reprod Open. 2025;2025:hoaf005. 10.1093/hropen/hoaf005. 39959763 10.1093/hropen/hoaf005 PMC11825388

[10] PCotASfR Medicine. Practice Committee of the American Society for Reproductive Medicine: Fertility Preservation in Patients Undergoing Gonadotoxic Therapy or Gonadectomy: A Committee Opinion. Fertil Steril. 2019;112:1022–33. 31843073 10.1016/j.fertnstert.2019.09.013

[11] Donnez J, Dolmans MM. Ovarian cortex transplantation: 60 reported live births brings the success and worldwide expansion of the technique towards routine clinical practice. J Assist Reprod Genet. 2015;32:1167–70. 10.1007/s10815-015-0544-9. 26210678 10.1007/s10815-015-0544-9 PMC4554373

[12] Donnez J, Dolmans MM, Demylle D, Jadoul P, Pirard C, Squifflet J, et al. Livebirth after orthotopic transplantation of cryopreserved ovarian tissue. Lancet. 2004;364:1405–10. 10.1016/S0140-6736(04)17222-X. 15488215 10.1016/S0140-6736(04)17222-X

[13] Roux C, Amiot C, Agnani G, Aubard Y, Rohrlich PS, Piver P. Live birth after ovarian tissue autograft in a patient with sickle cell disease treated by allogeneic bone marrow transplantation. Fertil Steril. 2010;93:2413.e2415–2419. 10.1016/j.fertnstert.2009.12.022. 10.1016/j.fertnstert.2009.12.022 20117783

[14] Dolmans MM, Hossay C, Nguyen TYT, Poirot C. Fertility preservation: how to preserve ovarian function in children, adolescents and adults. J Clin Med. 2021; 10. 10.3390/jcm10225247. 10.3390/jcm10225247 PMC8621487 34830528

[15] Demeestere I, Simon P, Dedeken L, Moffa F, Tsepelidis S, Brachet C, et al. Live birth after autograft of ovarian tissue cryopreserved during childhood. Hum Reprod. 2015;30:2107–09. 10.1093/humrep/dev128. 26062556 10.1093/humrep/dev128

[16] Segers I, Bardhi E, Mateizel I, Van Moer E, Schots R, Verheyen G, et al. Live births following fertility preservation using in-vitro maturation of ovarian tissue oocytes. Hum Reprod. 2020;35:2026–36. 10.1093/humrep/deaa175. 32829388 10.1093/humrep/deaa175

[17] Soler N, Cimadomo D, Escrich L, Grau N, Galan A, Alama P, et al. Rescue in vitro maturation of germinal vesicle oocytes after ovarian stimulation: the importance of the culture media. Hum Reprod. 2025;40:1504–15. 10.1093/humrep/deaf099. 40447125 10.1093/humrep/deaf099 PMC12314148

[18] Smith SD, Mikkelsen A, Lindenberg S. Development of human oocytes matured in vitro for 28 or 36 h. Fertil Steril. 2000;73:541–44. 10.1016/s0015-0282(99)00574-9. 10689010 10.1016/s0015-0282(99)00574-9

[19] Mulder RL, Font-Gonzalez A, Green DM, Loeffen EAH, Hudson MM, Loonen J, et al. Fertility preservation for male patients with childhood, adolescent, and young adult cancer: recommendations from the PanCareLIFE Consortium and the International Late Effects of Childhood Cancer Guideline Harmonization Group. Lancet Oncol. 2021;22:e57–e67. 10.1016/S1470-2045(20)30582-9. 33539754 10.1016/S1470-2045(20)30582-9

[20] Portela JMD, de Winter-Korver CM, van Daalen SKM, Meissner A, de Melker AA, Repping S, et al. Assessment of fresh and cryopreserved testicular tissues from (pre)pubertal boys during organ culture as a strategy for in vitro spermatogenesis. Hum Reprod. 2019;34:2443–55. 10.1093/humrep/dez180. 31858131 10.1093/humrep/dez180 PMC6936721

[21] Uijldert M, Meissner A, de Melker AA, van Pelt AMM, van de Wetering MD, van Rijn RR, et al. Development of the testis in pre-pubertal boys with cancer after biopsy for fertility preservation. Hum Reprod. 2017;32:2366–72. 10.1093/humrep/dex306. 29040511 10.1093/humrep/dex306

[22] Zielen AC, Peters KA, Shetty G, Gross DA, Hanna CB, Dovey SL, et al. Ultrasound-guided rete testis approach to sperm aspiration and spermatogonial stem cell transplantation in patients with azoospermia. Preprint at 10.1101/2025.03.25.25324518.

[23] Hermann BP, Sukhwani M, Winkler F, Pascarella JN, Peters KA, Sheng Y, et al. Spermatogonial stem cell transplantation into rhesus testes regenerates spermatogenesis producing functional sperm. Cell Stem Cell. 2012;11:715–26. 10.1016/j.stem.2012.07.017. 23122294 10.1016/j.stem.2012.07.017 PMC3580057

[24] Fayomi AP, Peters K, Sukhwani M, Valli-Pulaski H, Shetty G, Meistrich ML, et al. Autologous grafting of cryopreserved prepubertal rhesus testis produces sperm and offspring. Science. 2019;363:1314–19. 10.1126/science.aav2914. 30898927 10.1126/science.aav2914 PMC6598202

[25] Duffin K, Neuhaus N, Andersen CY, Barraud-Lange V, Braye A, Eguizabal C, et al. A 20-year overview of fertility preservation in boys: new insights gained through a comprehensive international survey. Hum Reprod open. 2024;2024:hoae010. 38449521 10.1093/hropen/hoae010 PMC10914450

[26] Barraud-Lange V, Boissel N, Gille AS, Jean C, Sitbon L, Schubert B, et al. A 10-year experience in testicular tissue cryopreservation for boys under 18 years of age: what can be learned from 350 cases? Andrology. 2024;12:385–95. 10.1111/andr.13493. 37418281 10.1111/andr.13493

[27] Cobo A, Cascante SD, García-Velasco J, Grifo JA. Is planned oocyte cryopreservation delivering? Reprod BioMed Online. 2025; 50:104794. 10.1016/j.rbmo.2025.104794 40287213

[28] Ho JR, Woo I, Louie K, Salem W, Jabara SI, Bendikson KA, et al. A comparison of live birth rates and perinatal outcomes between cryopreserved oocytes and cryopreserved embryos. J Assist Reprod Genet. 2017;34:1359–66. 28718080 10.1007/s10815-017-0995-2 PMC5633580

[29] Wang SF, Seifer DB. Age-related increase in live-birth rates of first frozen-thaw embryo versus first fresh transfer in initial assisted reproductive technology cycles without PGT. Reprod Biol Endocrinol. 2024;22. 42. 10.1186/s12958-024-01210-0. 38615016 10.1186/s12958-024-01210-0 PMC11015537

[30] Dolmans MM, von Wolff M, Poirot C, Diaz-Garcia C, Cacciottola L, Boissel N, et al. Transplantation of cryopreserved ovarian tissue in a series of 285 women: a review of five leading European centers. Fertil Steril. 2021;115:1102–15. 10.1016/j.fertnstert.2021.03.008. 33933173 10.1016/j.fertnstert.2021.03.008

[31] Missontsa MM, Bernaudin F, Fortin A, Dhedin N, Pondarre C, Yakouben K, et al. Ovarian tissue cryopreservation for fertility preservation before hematopoietic stem cell transplantation in patients with sickle cell disease: safety, ovarian function follow-up, and results of ovarian tissue transplantation. J Assist Reprod Genet. 2024;41:1027–34. 10.1007/s10815-024-03054-4. 38358434 10.1007/s10815-024-03054-4 PMC11052959

[32] Rives N, Courbière B, Almont T, Kassab D, Berger C, Grynberg M, et al. What should be done in terms of fertility preservation for patients with cancer? The French 2021 guidelines. Eur J Cancer. 2022;173:146–66. 10.1016/j.ejca.2022.05.013. 35932626 10.1016/j.ejca.2022.05.013

[33] Khattak H, Malhas R, Craciunas L, Afifi Y, Amorim CA, Fishel S, et al. Fresh and cryopreserved ovarian tissue transplantation for preserving reproductive and endocrine function: a systematic review and individual patient data meta-analysis. Hum Reprod update. 2022;28:400–16. 35199164 10.1093/humupd/dmac003 PMC9733829

[34] Donnez J, Dolmans M-M. Fertility preservation in women. N Engl J Med. 2017;377:1657–65. 29069558 10.1056/NEJMra1614676

[35] Moore HC, Unger JM, Phillips KA, Boyle F, Hitre E, Porter D, et al. Goserelin for ovarian protection during breast-cancer adjuvant chemotherapy. N Engl J Med. 2015;372:923–32. 10.1056/NEJMoa1413204. 25738668 10.1056/NEJMoa1413204 PMC4405231

[36] Turan V, Bedoschi G, Rodriguez-Wallberg K, Sonmezer M, Pacheco FS, Oktem O, et al. Utility of gonadotropin-releasing hormone agonists for fertility preservation: lack of biologic basis and the need to prioritize proven methods. J Clin Oncol. 2019;37:84–86. 30407897 10.1200/JCO.18.00420

[37] Su HI, Lacchetti C, Letourneau J, Partridge AH, Qamar R, Quinn GP, et al. Fertility preservation in people with cancer: ASCO guideline update. J Clin Oncol. 2025;43:1488–1515. 10.1200/JCO-24-02782. 40106739 10.1200/JCO-24-02782

[38] Nangia AK, Krieg SA, Kim SS. Clinical guidelines for sperm cryopreservation in cancer patients. Fertil Steril. 2013;100:1203–09. 10.1016/j.fertnstert.2013.08.054. 24182555 10.1016/j.fertnstert.2013.08.054

[39] Blecher GA, Chung E, Katz D, Kim SHK, Bailie J. Onco-testicular sperm extraction (oncotese): a contemporary concept review and report of Australian sperm retrieval rates and fertility outcomes. Urology. 2022;160:109–16. 10.1016/j.urology.2021.10.031. 34813838 10.1016/j.urology.2021.10.031

[40] Cirigliano L, Falcone M, Gul M, Preto M, Ceruti C, Plamadeala N, et al. Onco-TESE (Testicular Sperm Extraction): insights from a tertiary center and comprehensive literature analysis. Medicina. 2023;59:1226 10.3390/medicina59071226. 37512038 10.3390/medicina59071226 PMC10386487

[41] Abou-Mourad YR, Lau BC, Barnett MJ, Forrest DL, Hogge DE, Nantel SH, et al. Long-term outcome after allo-SCT: close follow-up on a large cohort treated with myeloablative regimens. Bone Marrow Transpl. 2010;45:295–302. 10.1038/bmt.2009.128. 10.1038/bmt.2009.128 19597425

[42] Barnes N, Chemaitilly W. Endocrinopathies in survivors of childhood neoplasia. Front Pediatr. 2014;2:101 10.3389/fped.2014.00101. 25295241 10.3389/fped.2014.00101 PMC4172013

[43] Borgmann-Staudt A, Rendtorff R, Reinmuth S, Hohmann C, Keil T, Schuster FR, et al. Fertility after allogeneic haematopoietic stem cell transplantation in childhood and adolescence. Bone Marrow Transpl. 2012;47:271–6. 10.1038/bmt.2011.78. 10.1038/bmt.2011.78 21478918

[44] Cedars MI. Managing poor ovarian response in the patient with diminished ovarian reserve. Fertil Steril. 2022;117:655–6. 10.1016/j.fertnstert.2022.02.026. 35367010 10.1016/j.fertnstert.2022.02.026

[45] EGGo POI, Webber L, Davies M, Anderson R, Bartlett J, Braat D, et al. ESHRE Guideline: management of women with premature ovarian insufficiency. Hum Reprod. 2016;31:926–37. 27008889 10.1093/humrep/dew027

[46] Molinari S, Parissone F, Evasi V, De Lorenzo P, Valsecchi MG, Cesaro S, et al. Serum anti-Müllerian hormone as a marker of ovarian reserve after cancer treatment and/or hematopoietic stem cell transplantation in childhood: proposal for a systematic approach to gonadal assessment. Eur J Endocrinol. 2021;185:717–28. 34519276 10.1530/EJE-21-0351

[47] Sarafoglou K, Boulad F, Gillio A, Sklar C. Gonadal function after bone marrow transplantation for acute leukemia during childhood. J Pediatr. 1997;130:210–6. 10.1016/s0022-3476(97)70345-7. 9042122 10.1016/s0022-3476(97)70345-7

[48] Sanders JE, Hawley J, Levy W, Gooley T, Buckner CD, Deeg HJ, et al. Pregnancies following high-dose cyclophosphamide with or without high-dose busulfan or total-body irradiation and bone marrow transplantation. Blood. 1996;87:3045–52. 8639928

[49] Chatterjee R, Goldstone AH. Gonadal damage and effects on fertility in adult patients with haematological malignancy undergoing stem cell transplantation. Bone Marrow Transpl. 1996;17:5–11. 8673055

[50] Diesch-Furlanetto T, Rovo A, Galimard JE, Szinnai G, Dalissier A, Sedlacek P, et al. Pregnancy and pregnancy outcomes after hematopoietic stem cell transplantation in childhood: a cross-sectional survey of the EBMT pediatric diseases working party. Hum Reprod. 2021;36:2871–82. 10.1093/humrep/deab199. 34529796 10.1093/humrep/deab199

[51] Korte E, Schilling R, Balcerek M, Byrne J, Dirksen U, Herrmann G, et al. Fertility-related wishes and concerns of adolescent cancer patients and their parents. J Adolesc Young- Adul. 2020;9:55–62. 10.1089/jayao.2019.0064 31621493

[52] Rotz SJ, Bjornard K, Hampanda K, Kumnick A, Maher JCY, Yu C, et al. Limited recommendations and evidence for timing and frequency of anti-mullerian hormone screening in female pediatric cancer survivors: a systematic review from the pediatric and adolescent committee of the oncofertility consortium. J Adolesc Young- Adult Oncol. 2025;14:212–9. 10.1089/jayao.2024.0111. 39552408 10.1089/jayao.2024.0111 PMC12223376

[53] Bjornard K, Close A, Rios J, Anazodo A, Levine J, Yu C et al. Current practices in the use of anti-mullerian hormone for surveillance of ovarian function in childhood cancer survivors: a report from the pediatric and adolescent committee of the oncofertility consortium. J Adolesc Young Adul 2025. 10.1177/21565333251359617. 10.1177/21565333251359617 40658204

[54] Jadoul P, Anckaert E, Dewandeleer A, Steffens M, Dolmans MM, Vermylen C, et al. Clinical and biologic evaluation of ovarian function in women treated by bone marrow transplantation for various indications during childhood or adolescence. Fertil Steril. 2011;96:126–133 e123. 10.1016/j.fertnstert.2011.03.108. 21550046 10.1016/j.fertnstert.2011.03.108

[55] Michel G, Socie G, Gebhard F, Bernaudin F, Thuret I, Vannier J, et al. Late effects of allogeneic bone marrow transplantation for children with acute myeloblastic leukemia in first complete remission: the impact of conditioning regimen without total-body irradiation-a report from the Société Française de Greffe de Moelle. J Clin Oncol. 1997;15:2238–46. 9196136 10.1200/JCO.1997.15.6.2238

[56] van Dorp W, Mulder RL, Kremer LC, Hudson MM, van den Heuvel-Eibrink MM, van den Berg MH, et al. Recommendations for Premature Ovarian Insufficiency Surveillance for Female Survivors of Childhood, Adolescent, and Young Adult Cancer: A Report From the International Late Effects of Childhood Cancer Guideline Harmonization Group in Collaboration With the PanCareSurFup Consortium. J Clin Oncol. 2016;34:3440–50. 10.1200/JCO.2015.64.3288. 27458300 10.1200/JCO.2015.64.3288 PMC5569686

[57] Meacham LR, Burns K, Orwig KE, Levine J. Standardizing risk assessment for treatment-related gonadal insufficiency and infertility in childhood, adolescent and young adult cancer: the pediatric initiative network risk stratification system. J Adolesc Young- Adult Oncol. 2020;9:662–6. 10.1089/jayao.2020.0012. 32456570 10.1089/jayao.2020.0012

[58] Wallace WH, Thomson AB, Kelsey TW. The radiosensitivity of the human oocyte. Hum Reprod. 2003;18:117–21. 10.1093/humrep/deg016. 12525451 10.1093/humrep/deg016

[59] Giorgiani G, Bozzola M, Cisternino M, Locatelli F, Gambarana D, Bonetti F, et al. Gonadal function in adolescents receiving different conditioning regimens for bone marrow transplantation. Bone Marrow Transpl. 1991;8:53. 1760641

[60] Lopez-Ibor B, Schwartz AD. Gonadal failure following busulfan therapy in an adolescent girl. Am J Pediatr Hematol Oncol. 1986;8:85–87. 3459380

[61] Singhal S, Powles R, Treleaven J, Horton C, Swansbury GJ, Mehta J. Melphalan alone prior to allogeneic bone marrow transplantation from HLA-identical sibling donors for hematologic malignancies: alloengraftment with potential preservation of fertility in women. Bone Marrow Transpl. 1996;18:1049–55. 8971372

[62] Green DM, Nolan VG, Goodman PJ, Whitton JA, Srivastava D, Leisenring WM, et al. The cyclophosphamide equivalent dose as an approach for quantifying alkylating agent exposure: a report from the Childhood Cancer Survivor Study. Pediatr Blood Cancer. 2014;61:53–67. 10.1002/pbc.24679. 23940101 10.1002/pbc.24679 PMC3933293

[63] Chow EJ, Stratton KL, Leisenring WM, Oeffinger KC, Sklar CA, Donaldson SS, et al. Pregnancy after chemotherapy in male and female survivors of childhood cancer treated between 1970 and 1999: a report from the Childhood Cancer Survivor Study cohort. Lancet Oncol. 2016;17:567–76. 10.1016/S1470-2045(16)00086-3. 27020005 10.1016/S1470-2045(16)00086-3 PMC4907859

[64] Rotz SJ, Hamilton BK, Wei W, Ahmed I, Winston SA, Ballard S, et al. Fertility potential and gonadal function in survivors of reduced-intensity hematopoietic stem cell transplantation. Transpl Cell Ther. 2024;30:534 e531–534 e513. 10.1016/j.jtct.2024.02.002. 10.1016/j.jtct.2024.02.002 PMC11056299 38342136

[65] George SA, Lai KW, Lewis RW, Bryson EW, Haight AE, Meacham LR. Comparison of anti-Mullerian hormone levels pre-and post-hematopoietic cell therapy in pediatric and adolescent females with sickle cell disease. Transplant Cell Ther. 2022;28:770.e1-770.e6. 10.1016/j.jtct.2022.08.014 35995392

[66] Alfraih F, Aljurf MD, Al Mohareb F, Alsharif F, Chaudhri N, Hanbali A, et al. Fertility recovery following allogeneic bone marrow transplantation in aplastic anemia: a study of 157 patients. Biol Blood Marrow Transpl. 2016;22:S181.

[67] Faraci M, Diesch T, Labopin M, Dalissier A, Lankester A, Gennery A, et al. Gonadal function after busulfan compared with treosulfan in children and adolescents undergoing allogeneic hematopoietic stem cell transplant. Biol Blood Marrow Transpl. 2019;25:1786–91. 10.1016/j.bbmt.2019.05.005. 10.1016/j.bbmt.2019.05.005 31082473

[68] Chatterjee R, Mills W, Katz M, McGarrigle HH, Goldstone AH. Germ cell failure and Leydig cell insufficiency in post-pubertal males after autologous bone marrow transplantation with BEAM for lymphoma. Bone Marrow Transpl. 1994;13:519–22. 8054905

[69] Cattoni A, Nicolosi ML, Capitoli G, Gadda A, Molinari S, Louka S, et al. Pubertal attainment and Leydig cell function following pediatric hematopoietic stem cell transplantation: a three-decade longitudinal assessment. Front Endocrinol. 2023;14:1292683. 10.3389/fendo.2023.1292683. 10.3389/fendo.2023.1292683 PMC10751351 38152128

[70] Lopez R, Plat G, Bertrand Y, Ducassou S, Saultier P, Berbis J, et al. Testosterone deficiency in men surviving childhood acute leukemia after treatment with hematopoietic stem cell transplantation or testicular radiation: an LEA study. Bone Marrow Transplant. 2021;56:1422–25. 33454725 10.1038/s41409-020-01180-y

[71] Sklar C. Reproductive physiology and treatment-related loss of sex hormone production. Med Pediatr Oncol. 1999;33:2–8. 10401490 10.1002/(sici)1096-911x(199907)33:1<2::aid-mpo2>3.0.co;2-7

[72] Meistrich ML, Wilson G, Brown BW, da Cunha MF, Lipshultz LI. Impact of cyclophosphamide on long-term reduction in sperm count in men treated with combination chemotherapy for Ewing and soft tissue sarcomas. Cancer. 1992;70:2703–12. 1423201 10.1002/1097-0142(19921201)70:11<2703::aid-cncr2820701123>3.0.co;2-x

[73] Centola GM, Keller JW, Henzler M, Rubin P. Effect of low-dose testicular irradiation on sperm count and fertility in patients with testicular seminoma. J Androl. 1994;15:608–13. 7721664

[74] Leiper A, Houwing M, Davies EG, Rao K, Burns S, Morris E, et al. Anti-mullerian hormone and inhibin B after stem cell transplant in childhood: a comparison of myeloablative, reduced intensity and treosulfan-based chemotherapy regimens. Bone Marrow Transpl. 2020;55:1985–95. 10.1038/s41409-020-0866-9. 10.1038/s41409-020-0866-9 32231250

[75] van Beek RD, Smit M, van den Heuvel-Eibrink MM, de Jong FH, Hakvoort-Cammel FG, van den Bos C, et al. Inhibin B is superior to FSH as a serum marker for spermatogenesis in men treated for Hodgkin’s lymphoma with chemotherapy during childhood. Hum Reprod. 2007;22:3215–22. 10.1093/humrep/dem313. 17981817 10.1093/humrep/dem313

[76] Preservation EGGoFF, Anderson RA, Amant F, Braat D, D’Angelo A, Chuva de Sousa Lopes SM, et al. ESHRE guideline: female fertility preservation. Hum Reprod Open. 2020;2020:hoaa052. 33225079 10.1093/hropen/hoaa052 PMC7666361

[77] Mulder RL, Font-Gonzalez A, Hudson MM, van Santen HM, Loeffen EAH, Burns KC, et al. Fertility preservation for female patients with childhood, adolescent, and young adult cancer: recommendations from the PanCareLIFE Consortium and the International Late Effects of Childhood Cancer Guideline Harmonization Group. Lancet Oncol. 2021;22:e45–e56. 10.1016/S1470-2045(20)30594-5. 33539753 10.1016/S1470-2045(20)30594-5

[78] Group EFfBW, Mitchell RT, Eguizabal C, Goossens E, Grynberg M, Jahnukainen K, et al. ESHRE good practice recommendations on fertility preservation involving testicular tissue cryopreservation in children receiving gonadotoxic therapies. Dagger Hum Reprod. 2025;40:1391–431. 10.1093/humrep/deaf106. 10.1093/humrep/deaf106 PMC12314154 40574354

[79] Frederick NN, Klosky JL, Meacham L, Quinn GP, Kelvin JF, Cherven B, et al. Fertility preservation practices at pediatric oncology institutions in the United States: a report from the Children’s Oncology Group. JCO Oncol Pract. 2023;19:e550–e558. 36763922 10.1200/OP.22.00349 PMC10113112

[80] Frederick NN, Klosky JL, Meacham LR, Quinn GP, Kelvin JF, Cherven B, et al. Infrastructure of fertility preservation services for pediatric cancer patients: a report from the Children’s Oncology Group. JCO Oncol Pract. 2022;18:e325–e333. 10.1200/OP.21.00275. 34709943 10.1200/OP.21.00275 PMC8932529

[81] Medicine PCotASfR. Fertility preservation in patients undergoing gonadotoxic therapy or gonadectomy: a committee opinion. Fertil Steril. 2019;112:1022–33. 31843073 10.1016/j.fertnstert.2019.09.013

[82] Pfitzer C, Orawa H, Balcerek M, Langer T, Dirksen U, Keslova P, et al. Dynamics of fertility impairment and recovery after allogeneic haematopoietic stem cell transplantation in childhood and adolescence: results from a longitudinal study. J Cancer Res Clin Oncol. 2015;141:135–42. 10.1007/s00432-014-1781-5. 25081929 10.1007/s00432-014-1781-5 PMC11823712

[83] von Wolff M, Imboden S, Stute P. Fertility preservation in females requiring gonadotoxic therapy should be more than freezing measures before therapy–secondary fertility preservation and menopause care management after therapy should also be considered. Arch Gynecol Obstet. 2025;312:1539–41. 40650693 10.1007/s00404-025-08104-5 PMC12589374

[84] Hyldgaard J, Bor P, Ingerslev HJ, Torring N. Comparison of two different methods for measuring anti-mullerian hormone in a clinical series. Reprod Biol Endocrinol. 2015;13:107. 10.1186/s12958-015-0101-5. 26394617 10.1186/s12958-015-0101-5 PMC4580367

[85] Signorello LB, Mulvihill JJ, Green DM, Munro HM, Stovall M, Weathers RE, et al. Congenital anomalies in the children of cancer survivors: a report from the childhood cancer survivor study. J Clin Oncol. 2012;30:239–45. 10.1200/JCO.2011.37.2938. 22162566 10.1200/JCO.2011.37.2938 PMC3269950

[86] Signorello LB, Cohen SS, Bosetti C, Stovall M, Kasper CE, Weathers RE, et al. Female survivors of childhood cancer: preterm birth and low birth weight among their children. J Natl Cancer Inst. 2006;98:1453–61. 10.1093/jnci/djj394. 17047194 10.1093/jnci/djj394 PMC2730161

[87] Henningsen AA, Opdahl S, Wennerholm UB, Tiitinen A, Rasmussen S, Romundstad LB, et al. Risk of congenital malformations in live-born singletons conceived after intracytoplasmic sperm injection: a Nordic study from the CoNARTaS group. Fertil Steril. 2023;120:1033–41. 10.1016/j.fertnstert.2023.07.003. 37442533 10.1016/j.fertnstert.2023.07.003

[88] Cobo A, Serra V, Garrido N, Olmo I, Pellicer A, Remohi J. Obstetric and perinatal outcome of babies born from vitrified oocytes. Fertil Steril. 2014;102:1006–1015 e1004. 10.1016/j.fertnstert.2014.06.019. 25064408 10.1016/j.fertnstert.2014.06.019

[89] Erden M, Uyanik E, Demeestere I, Oktay KH. Perinatal outcomes of pregnancies following autologous cryopreserved ovarian tissue transplantation: a systematic review with pooled analysis. Am J Obstet Gynecol. 2024;231:480–89. 10.1016/j.ajog.2024.04.012. 38621483 10.1016/j.ajog.2024.04.012 PMC11473709

[90] Xie Q, Jiang X, Zhao M, Xie Y, Fan Y, Suo L, et al. Effect of freezing and thawing on ejaculated sperm and subsequent pregnancy and neonatal outcomes in IVF. Front Endocrinol. 2024;15:1408662. 10.3389/fendo.2024.1408662. 10.3389/fendo.2024.1408662 PMC11684094 39736859

[91] Jahnukainen K, Tinkanen H, Wikstrom A, Dunkel L, Saarinen-Pihkala UM, Makinen S, et al. Bone marrow remission status predicts leukemia contamination in ovarian biopsies collected for fertility preservation. Leukemia. 2013;27:1183–5. 10.1038/leu.2012.279. 23079961 10.1038/leu.2012.279

[92] Chevillon F, Clappier E, Arfeuille C, Cayuela JM, Dalle JH, Kim R, et al. Minimal residual disease quantification in ovarian tissue collected from patients in complete remission of acute leukemia. Blood. 2021;137:1697–701. 10.1182/blood.2020007782. 33171484 10.1182/blood.2020007782

[93] Zver T, Frontczak S, Poirot C, Rives-Feraille A, Leroy-Martin B, Koscinski I, et al. Minimal residual disease detection by multicolor flow cytometry in cryopreserved ovarian tissue from leukemia patients. J Ovarian Res. 2022;15:9. 10.1186/s13048-021-00936-4. 35042558 10.1186/s13048-021-00936-4 PMC8767661

[94] Feraille A, Etancelin P, Troche S, Jardin F, Buchbinder N, Schneider P, et al. Detection of minimal residual disease in cryopreserved testicular tissue from (pre)pubertal boys with acute leukemia following first-line therapy. Hum Reprod. 2025;40:1476–84. 10.1093/humrep/deaf093. 40389239 10.1093/humrep/deaf093

[95] Sonmezer M, Sukur YE, Sacinti KG, Ozkavukcu S, Kankaya D, Atabekoglu CS, et al. Safety of ovarian cryopreservation and transplantation in patients with acute leukemia: a case series. Am J Obstet Gynecol. 2024;230:79 e71–79 e10. 10.1016/j.ajog.2023.08.032. 10.1016/j.ajog.2023.08.032 37666382

[96] Shapira M, Raanani H, Barshack I, Amariglio N, Derech-Haim S, Marciano MN, et al. First delivery in a leukemia survivor after transplantation of cryopreserved ovarian tissue, evaluated for leukemia cells contamination. Fertil Steril. 2018;109:48–53. 10.1016/j.fertnstert.2017.09.001. 29198847 10.1016/j.fertnstert.2017.09.001

[97] Sonmezer M, Ozkavukcu S, Sukur YE, Kankaya D, Arslan O. First pregnancy and live birth in Turkey following frozen-thawed ovarian tissue transplantation in a patient with acute lymphoblastic leukemia who underwent cord blood transplantation. J Assist Reprod Genet. 2020;37:2033–43. 10.1007/s10815-020-01850-2. 32556882 10.1007/s10815-020-01850-2 PMC7468030

[98] Soares M, Segers I, De Brucker M, Camboni A, Hossay C, Mateizel I et al. Cryopreserved ovarian tissue autotransplantation in an acute myeloid leukaemia survivor following extensive minimal residual disease screening: first reported live birth in Europe. J Assist Reprod Genet. 2025;42:1485–90. 10.1007/s10815-025-03447-z PMC12167415 40185953

[99] Rodriguez-Wallberg KA, Milenkovic M, Papaikonomou K, Keros V, Gustafsson B, Sergouniotis F, et al. Successful pregnancies after transplantation of ovarian tissue retrieved and cryopreserved at time of childhood acute lymphoblastic leukemia–a case report. Haematologica. 2021;106:2783. 34233451 10.3324/haematol.2021.278828 PMC8485665

[100] Chevillon F, Labrune E, Clappier E, Lapillonne H, Ballerini P, Lange VB et al. Ovarian tissue autotransplantation in acute leukemia: balancing the risk of relapse and the hope of parenthood. Haematologica 2025. 10.3324/haematol.2025.287942. 10.3324/haematol.2025.287942 PMC12951168 40637728

[101] Poirot C, Fortin A, Dhédin N, Brice P, Socié G, Lacorte J-M, et al. Post-transplant outcome of ovarian tissue cryopreserved after chemotherapy in hematologic malignancies. Haematologica. 2019;104:e360. 30765476 10.3324/haematol.2018.211094 PMC6669155

[102] Karavani G, Schachter-Safrai N, Revel A, Mordechai-Daniel T, Bauman D, Imbar T. In vitro maturation rates in young premenarche patients. Fertil Steril. 2019;112:315–22. 31056316 10.1016/j.fertnstert.2019.03.026

[103] Telfer EE, Andersen CY. In vitro growth and maturation of primordial follicles and immature oocytes. Fertil Steril. 2021;115:1116–25. 10.1016/j.fertnstert.2021.03.004. 33823993 10.1016/j.fertnstert.2021.03.004

[104] Hmaidan S, Van Heertum K, Bhatia S, Domm J, McManus M, Carroll C, et al. Safety and feasibility of fertility preservation and fertility outcomes in patients with sickle cell disease and transfusion-dependent beta thalassemia undergoing gene therapy. Transpl Cell Ther. 2025;31:934 e931–934 e938. 10.1016/j.jtct.2025.08.004. 10.1016/j.jtct.2025.08.004 40774509

[105] Ligon JA, Fry A, Maher JY, Foley T, Silbert S, Yates B, et al. Fertility and CAR T-cells: current practice and future directions. Transpl Cell Ther. 2022;28:605 e601–605 e608. 10.1016/j.jtct.2022.06.002. 10.1016/j.jtct.2022.06.002 PMC10332466 35705177

[106] Ligon JA, Cupit-Link MC, Yu C, Levine J, Foley T, Rotz S, et al. Pediatric cancer immunotherapy and potential for impact on fertility: a need for evidence-based guidance. Transpl Cell Ther. 2024;30:737–49. 10.1016/j.jtct.2024.06.006. 10.1016/j.jtct.2024.06.006 38866240

[107] Cosgrove C, Dellacecca ER, van den Berg JH, Haanen JB, Nishimura MI, Le Poole IC, et al. Transgenerational transfer of gene-modified T cells. J Immunother Cancer. 2019;7:186 10.1186/s40425-019-0657-2. 31307533 10.1186/s40425-019-0657-2 PMC6631543

[108] Salooja N, Szydlo RM, Socie G, Rio B, Chatterjee R, Ljungman P, et al. Pregnancy outcomes after peripheral blood or bone marrow transplantation: a retrospective survey. Lancet. 2001;358:271–6. 10.1016/s0140-6736(01)05482-4. 11498213 10.1016/s0140-6736(01)05482-4

[109] Marklund A, Nasiell J, Berger AS, Fagerberg A, Rodriguez-Wallberg KA. Pregnancy achieved using donor eggs in cancer survivors with treatment-induced ovarian failure: obstetric and perinatal outcome. J Women’s Health. 2018;27:939–45. 10.1089/jwh.2017.6703. 10.1089/jwh.2017.6703 PMC6159345 29715049

[110] Urbano MG. Tait DM. Can the irradiated uterus sustain a pregnancy? A literature review. Clin Oncol. 2004;16:24–8. 10.1016/s0936-6555(03)00199-7 14768752

[111] Critchley HO, Bath LE, Wallace WHB. Radiation damage to the uterus—review of the effects of treatment of childhood cancer. Hum Fertil. 2002;5:61–6. 10.1080/1464727022000198942 12082209

[112] Rodriguez-Wallberg KA, Olofsson JI. Future fertility in survivors of childhood cancer-examining the impact of cancer treatment on uterus function. Fertil Steril. 2019;111:262–3. 10.1016/j.fertnstert.2018.11.026. 30691627 10.1016/j.fertnstert.2018.11.026

[113] Beneventi F, Locatelli E, Giorgiani G, Zecca M, Locatelli F, Cavagnoli C, et al. Gonadal and uterine function in female survivors treated by chemotherapy, radiotherapy, and/or bone marrow transplantation for childhood malignant and non-malignant diseases. BJOG. 2014;121:856–65. 10.1111/1471-0528.12715. 24655331 10.1111/1471-0528.12715

[114] Courbiere B, Drikes B, Grob A, Hamidou Z, Saultier P, Bertrand Y, et al. The uterine volume is dramatically decreased after hematopoietic stem cell transplantation during childhood, regardless of the conditioning regimen. Fertil Steril. 2023;119:663–72. 36627013 10.1016/j.fertnstert.2022.12.040

[115] Saultier P, Guillerault L, Domenech C, Ducassou S, Gandemer V, Visentin S et al. Pregnancy outcomes in women conceiving after HSCT for childhood or adolescent leukemia. Blood. 2025;146:1056.10.1038/s41409-026-03019-442675121

[116] Chabut M, Schneider P, Courbiere B, Saultier P, Bertrand Y, Tabone M-D, et al. Ovarian function and spontaneous pregnancy after hematopoietic stem cell transplantation for leukemia before puberty: an LEA cohort study. Transplant Cell Ther. 2023;29:378. e371–378.e379. 10.1016/j.jtct.2023.02.019 36849077

[117] Khan Z, Gada RP, Tabbaa ZM, Laughlin-Tommaso SK, Jensen JR, Coddington CC III, et al. Unilateral oophorectomy results in compensatory follicular recruitment in the remaining ovary at time of ovarian stimulation for in vitro fertilization. Fertil Steril. 2014;101:722–7. 24355047 10.1016/j.fertnstert.2013.11.019

[118] Bjelland E, Wilkosz P, Tanbo T, Eskild A. Is unilateral oophorectomy associated with age at menopause? A population study (the HUNT2 Survey). Hum Reprod. 2014;29:835–41. 24549218 10.1093/humrep/deu026

[119] Yasui T, Hayashi K, Mizunuma H, Kubota T, Aso T, Matsumura Y, et al. Factors associated with premature ovarian failure, early menopause and earlier onset of menopause in Japanese women. Maturitas. 2012;72:249–55. 10.1016/j.maturitas.2012.04.002. 22572589 10.1016/j.maturitas.2012.04.002

[120] Thomas-Teinturier C, El Fayech C, Oberlin O, Pacquement H, Haddy N, Labbé M, et al. Age at menopause and its influencing factors in a cohort of survivors of childhood cancer: earlier but rarely premature. Hum Reprod. 2013;28:488–95. 23154067 10.1093/humrep/des391

[121] Bedrick BS, Kohn TP, Pecker LH, Christianson MS. Fertility preservation for pediatric patients with hemoglobinopathies: multidisciplinary counseling needed to optimize outcomes. Front Endocrinol. 2022;13:985525. 10.3389/fendo.2022.985525. 10.3389/fendo.2022.985525 PMC9638952 36353243

[122] SkordisN. Fertility and Pregnancy. In: Cappellini MD, Farmakis D, Porter J, Taher A, De Sanctis V, Subair S (editors). In: Proceedings of 2021 Guidelines: For the Management of Transfusion Dependent Thalassemia (TDT), 4th edn. Thalassemia International Federation©: Nicosia; 2023. 38683909

[123] Jarisch A, Germeyer A. Non-malignant diseases requiring stem cell transplantation. In: Fertility preservation in oncological and non-oncological diseases: a practical guide. Springer Nature Switzerland AG, 2020.

[124] Matthews M, Pollack R. Acute pain crisis in a patient with sickle cell disease undergoing ovarian stimulation for fertility preservation prior to curative stem cell transplantation: case report and literature review. J Assist Reprod Genet. 2017;34:1445–48. 10.1007/s10815-017-1008-1. 28801781 10.1007/s10815-017-1008-1 PMC5699988

[125] Pecker LH, Maher JY, Law JY, Beach MC, Lanzkron S, Christianson MS. Risks associated with fertility preservation for women with sickle cell anemia. Fertil Steril. 2018;110:720–31. 10.1016/j.fertnstert.2018.05.016. 30196969 10.1016/j.fertnstert.2018.05.016

[126] Schyrr F, Dolci M, Nydegger M, Canellini G, Andreu-Ullrich H, Joseph JM, et al. Perioperative care of children with sickle cell disease: a systematic review and clinical recommendations. Am J Hematol. 2020;95:78–96. 10.1002/ajh.25626. 31456233 10.1002/ajh.25626

[127] Sewaralthahab S, Alsubki LA, Alhrabi MS, Alsultan A. Effects of hydroxyurea on fertility in male and female sickle cell disease patients. A systematic review and meta-analysis. PLoS ONE. 2024;19:e0304241 10.1371/journal.pone.0304241. 38848387 10.1371/journal.pone.0304241 PMC11161076

[128] Nickel RS, Maher JY, Hsieh MH, Davis MF, Hsieh MM, Pecker LH. Fertility after curative therapy for sickle cell disease: a comprehensive review to guide care. J Clin Med. 2022;11:2318 10.3390/jcm11092318. 35566443 10.3390/jcm11092318 PMC9105328

[129] Loren AW, Chow E, Jacobsohn DA, Gilleece M, Halter J, Joshi S, et al. Pregnancy after hematopoietic cell transplantation: a report from the late effects working committee of the Center for International Blood and Marrow Transplant Research (CIBMTR). Biol Blood Marrow Transpl. 2011;17:157–66. 10.1016/j.bbmt.2010.07.009. 10.1016/j.bbmt.2010.07.009 PMC3017731 20659574

[130] Hudda Z, Lawhorn E, Mani B, Badia P, Frias O, Myers KC, et al. Longitudinal outcomes of fertility potential in female pediatric and young adult allogeneic hematopoietic stem cell transplant survivors. Transplant Cell Ther. 2025;31:S98.

[131] Sockel K, Neu A, Goeckenjan M, Ditschkowski M, Hilgendorf I, Kroger N, et al. Hope for motherhood: pregnancy after allogeneic hematopoietic cell transplantation (a national multicenter study). Blood. 2024;144:1532–42. 10.1182/blood.2024024342. 39007722 10.1182/blood.2024024342

[132] Mehta RS, Lee SJ, Gooley T, Thur L, Dahlberg A, Delaney C, et al. Long-term outcomes and quality of life with treosulfan-based conditioning in hematological malignancies. Blood Adv. 2025;9:2691–94. 10.1182/bloodadvances.2024015392. 39969207 10.1182/bloodadvances.2024015392 PMC12159895

[133] Weibring K, Lundberg FE, Cohn-Cedermark G, Rodriguez-Wallberg KA. Sperm quality in 1252 adolescents and young adults (AYAs) undergoing fertility preservation due to cancer or nonmalignant diseases. J Adolesc Young- Adul. 2025;14:68–76. 10.1089/jayao.2024.0068 39069896

